# Risk of mortality for small newborns in Brazil, 2011-2018: A national birth cohort study of 17.6 million records from routine register-based linked data

**DOI:** 10.1016/j.lana.2021.100045

**Published:** 2021-11

**Authors:** Enny S. Paixao, Hannah Blencowe, Ila Rocha Falcao, Eric O. Ohuma, Aline dos Santos Rocha, Flávia Jôse Oliveira Alves, Maria da Conceição N. Costa, Lorena Suárez-Idueta, Naiá Ortelan, Liam Smeeth, Laura C. Rodrigues, Joy E Lawn, Marcia Furquim de Almeida, Maria Yury Ichihara, Rita de Cássia Ribeiro Silva, Maria Gloria Teixeira, Mauricio L. Barreto

**Affiliations:** 1 Center for Data and Knowledge Integration for Health (CIDACS), Gonçalo Moniz Institute, Oswaldo Cruz Foundation, Salvador, Bahia, Brazil; 2 Maternal, Adolescent, Reproductive & Child Health (MARCH) Centre, London School of Hygiene & Tropical Medicine, London WC1E 7HT, UK; 3 Escola de Nutrição, Universidade Federal da Bahia, Salvador, Brazil; 4 Instituto de Saude Coletiva, Federal University of Bahia, Salvador, Bahia, Brazil; 5 Mexican Society of Public Health. Herschel 109, Anzures, Miguel Hidalgo, 11590, Mexico City; 6 Faculdade de Saude Publica, Universidade de São Paulo, Brazil

## Abstract

**Background:**

Preterm birth (<37 weeks), low birth weight (LBW,<2500g), and small for gestational age (SGA,<10th centile of birth weight for gestational age and sex) are markers of newborn vulnerability with a high risk of mortality. We estimated the prevalence of phenotypes combining these three markers and quantified the mortality risk associated with them.

**Methods:**

Population-based cohort study using routine register-based linked data on all births and deaths in Brazil from January 1, 2011, to December 31, 2018. We estimated the prevalence of preterm, LBW, and SGA individually and for phenotypes combining these characteristics. The mortality risk associated with each phenotype: early neonatal, late neonatal, neonatal, post-neonatal, infant, 1-4 years, and under five years was quantified using mortality rates and hazard ratios (HRs) with 95% confidence interval (CI) were estimated using Cox proportional hazard models.

**Findings:**

17,646,115 live births were included. Prevalence of preterm birth, LBW and SGA were 9.4%, 9.6% and 9.2%, respectively. Neonatal mortality risk was 16-fold (HR=15.9; 95% CI:15.7–16.1) higher for preterm compared to term, 3 times higher (HR=3.4; (95% CI:3.3–3.4) for SGA compared to adequate for gestational age (AGA), and >25 times higher for LBW (HR=25.8; (95% CI:25.5-26.1) compared to normal birth weight (NBW). 18% of all live births were included in one of the small vulnerable newborn phenotypes. Of those 8.2% were term-SGA (4.7%NBW, 3.5%LBW), 0.6% were term-AGA-LBW, 8.3% preterm-AGA (3.8%NBW, 4.5%LBW) and 1.0% preterm-SGA-LBW. Compared to term-AGA-NBW, the highest mortality risk was for preterm-LBW phenotypes (HR=36.2(95%CI 35.6-36.8) preterm-AGA-LBW, HR=62.0(95%CI 60.8-63.2) preterm-SGA-LBW). The increased mortality risk associated with vulnerable newborn phenotypes was highest in the first month of life, with attenuated but continued high risk in the post-neonatal period and 1-4 years of age.

**Interpretation:**

Our findings support the value of using more detailed phenotypes to identify those at highest risk. More granular data can inform care at the individual level, advance research, especially for prevention, and accelerate progress towards global targets such as the Sustainable Development Goals.

**Funding:**

Wellcome Trust

**Research in context**

**Evidence before this study**

Previous studies have shown the increased risk of neonatal and infant mortality among low birth weight (LBW, birth weight below 2500g), preterm (birth before 37 gestational weeks) and SGA (<10th centile of birth weight for gestational age and sex) infants. However, these studies assume homogeneity within each of these groups even though substantial differences may exist and that babies can be classified in more than one group. For the Lancet Vulnerable Newborn series, Ashorn et al. (2020) recently highlighted the importance of closing gaps in knowledge and defined detailed vulnerable newborn phenotypes by combining preterm, SGA, and LBW.

**Added value of this study**

In this population-based study of nearly 17.6 million live births, we estimated that prevalences of preterm birth, SGA and LBW individually were around 9%. However, 18% of all live births were included in one of the small vulnerable newborn phenotypes, and 1% were simultaneously preterm, LBW and SGA. These newborns were the most vulnerable, with the highest mortality risk 62 times greater compared to term babies who were not LBW or SGA. The increased mortality risks associated with preterm birth, SGA and LBW were most marked in the first month of life, however, some increased risk remained up to 1-4 years.

**Implications of all the available evidence**

To our knowledge, this analysis combining the three phenotypes (namely preterm birth, SGA and LBW) as proposed has not been presented before. The proposed approach provides more granular information by moving beyond simplistic dichotomous cut-offs, which could better support research on causal pathways and mechanisms to distinguish between the several phenotypes. The great variety observed in the mortality risk highlights the importance of this work, enabling better monitoring and management of small vulnerable newborns, optimising interventions, and enhancing resource allocation, delivering preventive and curative procedures and programming for those most in need. Therefore, the findings of this study are of public health importance and have the potential to contribute towards faster progress to achieving Sustainable Development Goals (SDGs) and Global Nutrition targets.

Alt-text: Unlabelled box

## 1 Introduction

Preterm birth (birth before 37 gestational weeks), small for gestational age (SGA, <10th centile of birth weight for gestational age and sex) (a proxy of intrauterine growth restriction), and low birth weight (LBW, birth weight below 2500g) are markers of newborn vulnerability with short and long-term outcomes [1]. LBW can be due to being preterm and/or intrauterine growth restriction. Each year it is estimated that 20.5 million newborns are born LBW - 15.5% of all births worldwide, however, around 80% of neonatal deaths occur in this group. Newborns who are both preterm and small for gestational age (SGA) are at 15 times greater mortality risk than those born at term and with appropriate size for gestational age (AGA) (between 10^th^ and 90^th^ centile) and sex [[2], [3], [4], [5], [6]]. In addition to increased mortality risk, small size at birth is associated with increased neonatal morbidity, childhood stunting and developmental delays, with lifelong consequences, including chronic health disorders and reduced human capital [[7], [8], [9]].

LBW has often been used as an indicator of newborn susceptibility; however, in general, most countries’ routine statistical data do not distinguish between newborns with intrauterine growth restriction (IUGR) (typically defined by the proxy, SGA) from those born preterm. Although there are national (and hence regional and global) estimates for the prevalence of preterm birth [12], LBW [5,10], there are much fewer comparable data on SGA with limited estimates for low- and middle-income countries based on only 23 studies [6]. There is a lack of data combining vulnerability of newborns using these three parameters (preterm birth, LBW, and SGA) to give more detailed phenotypes.

Previous studies have shown the increased risk of neonatal and infant mortality among LBW, preterm and SGA infants [2,11]. However, these studies assume homogeneity within each of these groups even though substantial differences may exist, and babies can fall in more than one group. For the Lancet Vulnerable Newborn series, Ashorn et al. (2020) recently highlighted the importance of closing gaps in knowledge and defined detailed vulnerable newborn phenotypes by combining preterm, SGA, and LBW [12]. A consortium is working on multi-country analyses to inform estimates of vulnerable newborn phenotypes and mortality population attributable risk [12]. This study uses national Brazilian linked data on more than 17 million live births over seven years. It aims to:

1 Estimate the prevalence of preterm, LBW, and SGA separately and for the detailed vulnerable newborn phenotypes.
2 Quantify the mortality risk (early neonatal, late neonatal, neonatal, post-neonatal, infant mortality, and under five years mortality) for each defined small vulnerable newborn phenotype.

Findings will be useful to inform policies and individual level care, advance research, especially for prevention, and accelerate progress towards global targets, such as the Sustainable Development Goals (SDGs) on ending preventable newborn and child deaths by 2030 and the WHO global nutrition targets for 2025, which include a 30% reduction in LBW [13,14].

## 2 Methods

We conducted a population-based electronic cohort study by linking routine data on live births with records of deaths in Brazil from January 1, 2011, to December 31, 2018. The data consist of live births followed up until the age of five years old, death or up to December 31, 2018 – whichever was first.

### 2.1 Data sources

We obtained data from the national Live Birth Information System- SINASC (Sistema de Informação sobre Nascidos Vivos), and national Mortality Information System-SIM (Sistema de Informação sobre Mortalidade) in Brazil from 2011-2018.

The SINASC is an information system that records data from the Declaration of Live Birth, a legal document completed by the health worker who attends the delivery. It covers over 98% of the Brazilian territory [15] and includes information on the mother (e.g., maternal age, schooling, marital status, and ethnicity); pregnancy information (e.g., antenatal appointments, length of gestation, multiple fetuses); and information on the newborn (e.g., birth weight, sex) [16].

Death-related information was obtained from SIM where all deaths are recorded. SIM includes information on the deceased and on the deceased's mother. As of 2011, SIM was estimated to cover over 97% of all deaths in Brazil [15].

### 2.3 Linkage process

Since there is no unique identifier in the Brazilian Information System, we linked SINASC live births records with deaths registered in SIM using the name of the mother, maternal date of birth or age (when date of birth was missing), and the municipality of residence of the mother as matching variables. The linkage was performed with CIDACS-RL-Record Linkage [17], a novel record-linkage tool developed to link large-scale administrative Brazilian datasets. Linkage procedures were conducted at the centre in a strict data protection environment and according to ethical and legal rules [18]. CIDACS-RL applies the combination of indexing and searching algorithms implemented in Apache Lucene solution as the blocking strategy. The indexation strategy allows the CIDACS-RL to search the most similar records from the Indexed SIM for each record in SINASC and submit them to the pairwise comparisons step. Candidate linking records are ordered by the scores, and only the comparison pair with the highest score is retained as a potential link. All remaining candidate records are discarded. A sample of 2000 pairs stratified in three categories of linkage score (high score – above 0.95, intermediate score – values between 0.90 and 0.95, and low score - below 0.90) was obtained and manually reviewed to evaluate the linkage quality. In this validation process, we obtained a mean sensitivity and specificity of over 93%.

### 2.4 Data processing, exclusions, and definitions

According to the SINASC guideline, gestational age is determined using either ultrasound, last menstrual period or clinical examination and the birth weight should be measured within 5 hours after the birth. Once the data were linked, we excluded records with a missing gestational age at birth, birth weight, or newborn sex for whom it is not possible to assess size for gestational age. We also excluded implausible birth weights defined as <350g or ≥ 6500g. Low birth weight was defined as birth weight < 2500 g, normal birth weight (NBW) as birthweight ≥2500g and <6500g, preterm birth as a gestational age < 37 completed weeks, term as gestational age ≥37 weeks and <42 weeks, and small for gestational age (SGA) defined as <10^th^ centile for birth weight for gestational age in completed weeks at birth by sex using the INTERGROWTH-21^st^ international newborn size standards [19] and appropriate size for gestational age (AGA) between 10th and 90th centiles. The INTERGROWTH-21^st^ international newborn size standards are from 24 to <43 weeks gestational age, and therefore we also excluded observations <24 weeks or ≥43 weeks at birth [19]. Those classified as large for gestational age (above the 90^th^ centile using the INTERGROWTH-21^st^) were excluded since this paper focuses on small newborns, and large babies will be studied in a subsequent analysis.

After all exclusions, live births were classified according to mutually exclusive phenotypes based on combinations of LBW, preterm birth and SGA [12]. The phenotypes are: term+ AGA+ NBW; term+SGA+NBW; preterm+AGA+NBW; term+AGA+LBW; term+SGA+LBW; preterm+AGA+LBW; and preterm+SGA+LBW. Term+AGA+NBW was used as the reference group. We further reclassified phenotypes into a binary variable: those not small (term +AGA+NBW) and those small (all remaining phenotypes).

We classified mortality as early neonatal (from birth up to 6 days), late neonatal (7-27 days), neonatal (from birth up to 27 days), post-neonatal (28–364 days), infant mortality (birth to 364 days), mortality between one to four years (after the first year of life up to five years) and under-five mortality (from birth up to five years).

### 2.5 Statistical analyses

We estimated the prevalences of preterm, SGA and LBW individually and each combined vulnerable newborn phenotype. Descriptive statistics are presented for maternal sociodemographic data and newborn characteristics. Mortality rates (deaths/1,000 person-year (PY) and crude hazard ratios (HRs) with 95% were estimated using Cox regression. Kaplan-Meier curves were plotted to compare the mortality by phenotype. We adjusted for maternal age, maternal race/ethnicity, marital status, maternal education, sex of newborn, multiple pregnancy, presence of congenital abnormalities detected at birth, and mode of delivery. We also conducted a sensitivity analysis, excluding newborn with congenital abnormalities. Data analyses were performed in Stata version 15.0.

Ethical approval was obtained from the Federal University of Bahia's Institute of Public Health Ethics Committee (CAAE registration number: 18022319.4.0000.5030).

### 2.6 Role of the Funding Source

Wellcome Trust (205377/Z/16/Z). ESP is funded by the Wellcome Trust Grant number 213589/Z/18/Z; ASR is funded by the Brazilian Federal Agency for Support and Evaluation of Graduate Education (CAPES). FJOA is funded by the Bahia Research Support Foundation. This research was funded by the Wellcome Trust [Grant number; 202912/Z/16/Z]. NIHR Grant number 122844. The funders had no role in study design, analysis, decision to publish or preparation of the manuscript.

## 3 Results

**Figure 1:**
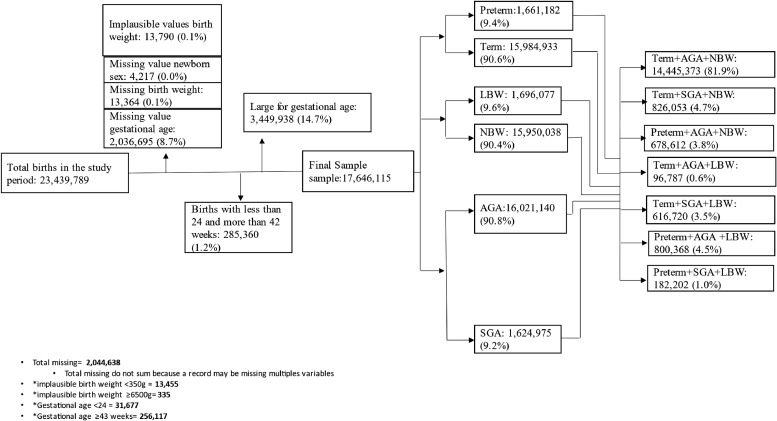
Study population.

During the study period, from January 1^st^ 2011, to December 31 2018, 23,439,789 live births were registered in SINASC. Of these, 5,793,674 (24.7%) were excluded, 2,044,638 (8.7%) due to missing data, 13,790 (0.1%) due to implausible birth weight values, 285,360 (1.2%) gestational age <24 weeks or ≥43 weeks at birth and 3,449,938 (14.7%) large for gestational age (Figure 1). A higher percentage of missing information on core variables (birth weight, gestational age at birth and newborn sex) were observed among live births of mixed race and indigenous, single, less educated mothers (supplementary Table S2).

Overall, 9.4% of all included live births were born preterm, 9.2% were SGA, and 9.6% were LBW. Over 40% of all preterm births were AGA and born with a normal birth weight (preterm+AGA+NBW: 3.8% of all live births), while 4.5% of all live births were preterm, AGA and LBW. The prevalence of LBW combined with SGA among term live births was 3.5%, and 0.6% of live births were term, LBW and AGA. The prevalence of term, SGA, and NBW was 4.7%, and 1% of all births were preterm, SGA, and LBW newborns (Figure 1).

In general, the prevalence of small live births (those with at least one of the characteristics i.e., preterm birth, SGA and LBW) was higher in mothers younger than 20 years (n=675,571, 20.7%) and older than 35 years (n =451,445, 20.3%), among indigenous and black (n= 27,233, 22.2%; 192,615, 20.2% respectively), widows and single mothers (n= 5,983, 19.4%; n =1,406,328, 19.0% respectively), and less educated women (n =24,623, 25.4%). About 38% of the small vulnerable babies had a congenital abnormality (Table 1). The characteristics of live births by each of the phenotypes are presented in supplementary Table S1.

**Table 1:** Characteristics of 17,646,115 delivering live birth included in this study in Brazil from 2011-2018 by vulnerability status.

Characteristics	Not Small live births*	Small live births⁎⁎	Total births	Prevalence of Small live births
**Maternal age (years)**				
<20 years	2,592,357	675,571	3,267,928	20.7
20-35	10,085,172	2,073,674	12,158,846	17.1
>35	1,767,728	451,445	2,219,173	20.3
**Maternal ethnicity**				
White	5,251,965	1,107,267	6,359,232	17.4
Black	761,388	192,615	954,003	20.2
Asian	58,165	12,696	70,861	17.9
Mixed race	7,700,328	1,719,443	9,419,771	18.3
Indigenous	98,233	27,994	126,227	22.2
**Marital status**				
Single	5,977,909	1,406,328	7,384,237	19.0
Married/union	8,132,186	1,718,753	9,850,939	17.4
Widow	24,936	5,983	30,919	19.4
Divorced	156,961	35,497	192,458	18.4
**Maternal education**				
None	72,304	24,623	96,927	25.4
1-3 years	403,719	111,272	514,991	21.6
4-7 years	2,646,788	667,899	3,314,687	20.1
8-12 years	8,436,419	1,812,841	10,249,260	17.7
≥ 12 years	2,683,797	537,768	3,221,565	16.7
**Multiples fetuses**				
Yes	108,573	312,882	421,455	74.2
None	14,316,826	2,883,468	17,200,294	16.8
**Sex of newborn**				
Male	7,378,763	1,647,549	9,026,312	18.3
Female	7,066,610	1,553,193	8,619,803	18.0
**Congenital abnormalities**				
Yes	91,520	55,997	147,517	38.0
None	14,022,232	3,067,722	17,089,954	18.0
**Mode of delivery**				
Caesarean section	7,947,149	1,745,066	9,692,215	18.0
Vaginal Delivery	6,482,878	1,452,186	7,935,064	18.3
**Neonatal deaths**				
Yes	26,140	93,366	119,506	78.1
No	14,378,412	3,070,718	17,449,130	17.6
**Early Neonatal deaths**				
Yes	17,885	68,472	86,357	79.3
No	14,378,412	3,070,718	17,449,130	17.6
**Late Neonatal deaths**				
Yes	8,255	24,894	33,149	75.1
No	14,378,412	3,070,718	17,449,130	17.6
**Post Neonatal deaths**				
Yes	25,209	29,727	54,936	54.1
No	14,378,412	3,070,718	17,449,130	17.6
**Infant Mortality**				
Yes	51,349	123,093	174,442	70.6
No	14,378,412	3,070,718	17,449,130	17.6
**Under five mortality**				
Yes	65,348	129,489	194,837	66.5
No	14,378,412	3,070,718	17,449,130	17.6
**1-4 mortality**				
Yes	13,999	6,396	20,395	31.4
No	14,378,412	3,070,718	17,449,130	17.6

⁎ Not Small live births=T+AGA+NBW,

⁎⁎ Small live births=all remain phenotypes In view of the very large sample size in our dataset, all between group comparisons are highly significant with very low p-values.

The mortality risk was higher in the early neonatal period, with nearly three-quarters (72.3%) of all neonatal deaths and 44.3% of all under-five deaths occurring in this period (n=86,357) (Table 1). Mortality risk decreased substantially in the post-neonatal and 1–4-year periods but remained elevated for all small phenotypes compared to ‘non-small’ peers. The overall adjusted neonatal mortality risk was over 15 times greater among preterm when compared to term newborns (HR=15.9; 15.7-16.1), 3-fold among SGA when compared to AGA (HR=3.4; 3.3-3.4), and 25-fold among LBW when compared to NBW (HR=25.8; 25.5-26.1) live births (Table 2).

**Table 2:** Mortality risk by age group associated with preterm birth, small for gestational age (SGA) and low birth weight (LBW), Brazil 2011-2018.

Mortality risk	Preterm birth*			SGA#		LBW$	
	HR (95%CI)			HR (95%CI)		HR (95%CI)	
	Unadjusted		Adjusted	Unadjusted	Adjusted	Unadjusted	Adjusted
**Early Neonatal Mortality (birth to 6 days)**	19.9 (19.6-20.1)		17.3 (17.1-17.6)	4.4 (4.3-4.4)	3.4 (3.3-3.4)	30.5 (30.1-30.9)	27.8 (27.4-28.2)
**Late Neonatal Mortality (7-27 days)**	14.1 (13.8-14.4)		12.6 (12.3-12.9)	4.1 (4-4.2)	3.3 (3.2-3.4)	22.7 (22.2-23.1)	21.0 (20.5-21.5)
**Post Neonatal Mortality (28-364 days)**	5.1 (5-5.2)		4.4 (4.4-4.5)	3.4 (3.3-3.4)	2.7 (2.7-2.8)	8.2 (8.1-8.3)	7.3 (7.2-7.4)
**1-4 years (≥1 -** <**5 years)**	1.9 (1.8-1.9)		1.7 (1.7-1.8)	2.0 (1.9-2.1)	1.8 (1.7-1.9)	2.6 (2.5-2.6)	2.4 (2.3-2.5)
**Neonatal Mortality (birth to 28 days)**	18.2 (18-18.4)	15.9 (15.7-16.1)		4.3 (4.2-4.4)	3.4 (3.3-3.4)	28.2 (27.9-28.5)	25.8 (25.5-26.1)
**Infant Mortality (birth to 364 days)**	12.2 (12.1-12.3)	10.6 (10.5-10.7)		4.0 (4.0-4.0)	3.2 (3.1-3.2)	19.1 (18.9-19.2)	17.2 (17.1-17.4)
**Under five mortality (birth up to 5 years)**	10.3 (10.2-10.4)	9.0 (8.9-9.0)		3.8 (3.7-3.8)	3.0 (3.0-3.0)	15.8 (15.7-15.9)	14.3 (14.2-14.4)

⁎ Reference group 37+,

$ Reference group 2500+,

# Reference group Adequate for gestational age.

Figure 3 shows Kaplan-Meier curves for the likelihood of death in the study population from birth up to five years. The probability of death increased rapidly during the neonatal period. The highest risk is observed among preterm+SGA+LBW newborns, with 12% dying before the 28^th^ day of life and 16% dying before five years. Among preterm+AGA+LBW there was also a high probability of death at around 6% during the neonatal period and 7% dying within five years.

**Figure 2:**
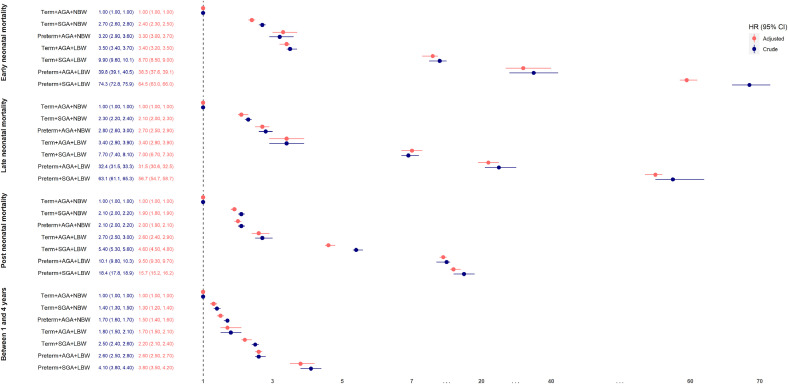
Cox proportional hazards for mortality risk comparing the different phenotypes.

The mortality risk varied markedly from one phenotype to another. Higher mortality risks were seen in those phenotypes, including more than one feature of preterm, SGA and LBW. Live births at term, SGA, with NBW were 2.3 (95% IC 2.2-2.4) more likely to die than term, AGA and NBW (Table 3). The neonatal mortality risk was substantially higher among live births with phenotypes that combined preterm birth and LBW, i.e., adjusted neonatal mortality was 36-fold among preterm, AGA live births that were LBW (HR= 36.2; 95% IC 35.6-36.8) and was 62-fold among those preterm, SGA and LBW (HR= 62.0; 95% IC 60.8-63.2) compared to term, adequate for gestational age and NBW. Infant and under-five mortality were 23-fold and 19-fold higher among preterm, AGA live births that were LBW and 39-fold and 32-fold higher among those preterm, SGA and LBW compared to term, AGA and NBW (Table 3). The relative mortality risk fell substantially for post-neonatal and 1–4-year, 9-fold and 2.6-fold among preterm, AGA live births that were LBW and 16-fold and 4-fold among those preterm, SGA and LBW compared to term, AGA and NBW (Figure 2). The analysis restricted to newborns without congenital abnormalities showed slightly lower relative mortality for most of the phenotypes and a higher relative mortality for the smallest newborns (preterm+AGA+LBW and preterm+SGA+LBW) (supplementary Table 3).

**Table 3:** Mortality risk by age group associated with the small vulnerable newborn phenotypes, Brazil 2011-2018.

	Neonatal mortality				Infant mortality				Under five mortality			
	HR (95%CI)				HR (95%CI)				HR (95%CI)			
	Number of deaths	Deaths/1000 PY	Unadjusted	Adjusted	Number of deaths	Deaths/1000 PY	Unadjusted	Adjusted	Number of deaths	Deaths/1000 PY	Unadjusted	Adjusted
**Term+AGA+NBW**	26140	24.6 (24.3-24.9)	Ref	Ref	51349	3.8 (3.8-3.9)	Ref	Ref	65348	1.4 (1.4-1.4)	Ref	Ref
**Term+SGA+NBW**	3800	62.8 (60.8-64.8)	2.5 (2.5-2.6)	2.3 (2.2-2.4)	6842	8.9 (8.7-9.1)	2.3 (2.3-2.4)	2.1 (2-2.1)	8188	3.0 (2.9-3.0)	2.2 (2.1-2.2)	2.0 (1.9-2.0)
**Preterm+AGA+NBW**	4053	81.5 (79.1-84.1)	3.3 (3.2-3.4)	3.2 (3.1-3.3)	6520	10.3 (10.1-10.6)	2.7 (2.6-2.8)	2.6 (2.5-2.7)	7444	3.3 (3.2-3.4)	2.4 (2.4-2.5)	2.3 (2.3-2.4)
**Term+AGA+LBW**	572	80.7 (74.4-87.6)	3.3 (3.0-3.6)	3.4 (3.1-3.7)	1031	11.6 (10.9-12.3)	3.0 (2.8-3.2)	3.0 (2.8-3.2)	1195	3.8 (3.6-4.0)	2.7 (2.6-2.9)	2.7 (2.6-2.9)
**Term+SGA+LBW**	10181	227.4 (223.1-231.9)	9.2 (9.0-9.4)	8.2 (8-8.4)	15903	28.2 (27.8-28.7)	7.3 (7.2-7.5)	6.4 (6.3-6.5)	17395	8.6 (8.5-8.7)	6.3 (6.2-6.4)	5.5 (5.4-5.6)
**Preterm+AGA+LBW**	52663	945.7 (937.7-953.8)	37.5 (36.9-38)	36.2 (35.6-36.8)	65662	95.6 (94.8-96.3)	24.1 (23.9-24.4)	23.1 (22.8-23.4)	67517	28.1 (27.8-28.3)	19.6 (19.4-19.8)	18.8 (18.6-19.0)
**Preterm+SGA+LBW**	22097	1829.9 (1805.9-1854.2)	70.8 (69.6-72.1)	62.0 (60.8-63.2)	27135	186.3 (184.1-188.5)	45.6 (44.9-46.2)	39.3 (38.6-39.9)	27750	54.2 (53.6-54.8)	36.9 (36.4-37.4)	31.9 (31.5-32.4)

**Figure 3:**
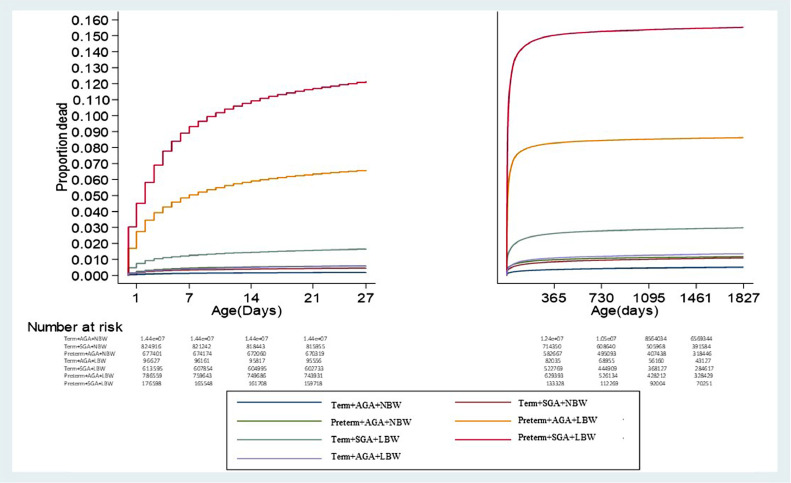
Figure 2, Figure 3

## 4 Discussion

In this population-based study of nearly 17.6 million live births over eight years in Brazil the prevalences of preterm birth, SGA and LBW were around 9%. The mortality associated with being born preterm or LBW was higher than for SGA. We estimated that 18% of all live births were included in one of the small vulnerable newborn phenotypes. While only 1% were simultaneously preterm, LBW and SGA, these newborns were the most vulnerable, with the highest mortality risk at 62-fold compared to term babies who were not LBW or SGA. As the preterm-LBW combination is likely to be a proxy of lower gestational ages, this finding further supports the importance of gestational age in neonatal mortality risk. The increased mortality risks associated with preterm birth, SGA and LBW were most marked in the first month of life, but some increased risk remained up to 1-4 years.

The elevated all-cause and cause-specific mortality risk associated with preterm birth is well known in both high-income [11,[20], [21], [22], [23], [24], [25], [26]] countries and from LMIC datasets [2,6,27]. The higher risk for neonates who are both preterm and SGA has also been described in several papers [2,6,27,28]. However, to our knowledge, this analysis combining the three phenotypes (preterm birth, SGA and LBW) as proposed by Ashorn et al. (2020) [12] has not been presented before. By moving beyond the simplistic dichotomous cut-offs, the proposed approach provides more granular information which could better support research on causal pathways and mechanisms to distinguish between the several phenotypes [12]. The great variety observed in the mortality risk highlights the importance of this work. It should enable better monitoring and management of small vulnerable newborns, optimising interventions, and enhancing resource allocation, delivering preventive and curative procedures and programming for those most in need. Therefore, the findings of this study are of public health importance and have the potential to contribute to faster progress towards Sustainable Development Goals (SDGs) and Global Nutrition targets [13,14,29].

Most LMIC studies on mortality risk associated with preterm births, intrauterine growth restriction and LBW include neonatal deaths and sometimes infant deaths [2,6,27], with more limited data on long-term sequelae [30]. Although in our cohort over 61% of deaths occurred in the neonatal period (first 28 days), an increased mortality risk persisted into early childhood and at least up to 5 years of age, as previously described [26]. Longer follow-up would be important to estimate the risk during late childhood, adolescence, and adulthood.

Our study has strengths and weaknesses. This population-based cohort has a very large sample size with sufficient power to assess mortality even for lower prevalence phenotypes. We also had information on covariates, which enabled control for several potential confounders. There are, however, limitations. First, the missing data were not distributed randomly: for example, a higher percentage of missing information was observed among more vulnerable groups, such as indigenous and less educated women. Since risks of small size at birth and associated mortality are likely to be higher in these vulnerable populations, this may add biases. Secondly the preterm births data in SINASC-Brazil have previously been found to underestimate the population preterm birth rate by 15% [31]. Third, the linkage process to identify deaths could have introduced classification bias due to linkage errors (missed matches or false matches). However, such linkage errors are probably non-differential, so misclassification of deaths should not vary according to gestational age or birth weight, and are therefore unlikely to introduce bias in the estimated hazard ratio, although the absolute measures of risk may be underestimated. Fourth, residual confounding is possible because we only had a limited number of possible confounders to analyse, and data on maternal comorbidities, quality or type of obstetric care might ideally have been adjusted for. Finally, we did not explore the duration of gestation and the birth weight other than classifying newborns into dichotomous variables. However, as previous research has shown, mortality varies markedly, very steep for lower gestational ages and birth weight. In future analyses it would be important to further improve our understanding of these cut-offs for mortality prediction purposes, including analysing using continuous interaction variables. Finally, although we conducted a sensitivity analyses excluding the group with congenital abnormalities an investigation focused exclusively on this group should be conducted.Ultimately SGA was not as predictive of mortality as preterm and low birthweight, and these more granular phenotypes better illuminate the variation in risk. Such evidence can inform research on causal pathways and help to better target interventions, such as early detection of risk, improved hospital based small and sick newborn care linked to comprehensive child nutrition and health, all of which can improve the survival of small and vulnerable newborns in LMICs and guide progress towards a decline in child mortality.

## Contributors

ESP, HB, JEL, EOO, MLB developed the study concept. MLB, MYI, MGT acquired the data. ESP, IRF, ASR, FJOA, MCC, LSI, NO, LS, LCR, MFA, RCRS contributed to interpretation of results. ESP wrote the first draft. All authors decided to publish and revised the manuscript and approved the final version.

## Declaration of Interests

We declare no competing interests.

This research was funded in part by the Wellcome Trust. For the purpose of open access, the author has applied a CC BY public copyright licence to any Author Accepted Manuscript version arising from this submission.

## Data sharing

All data supporting the findings presented here were obtained from Centro de Integração de Dados e Conhecimentos para Saúde (CIDACS). It is important to note that restrictions apply to the availability of these data. However, upon reasonable request and provided all ethical and legal requirements are met, the institutional data curation team can make the data available. Information on how to apply for access to the data can be found at https://cidacs.bahia.fiocruz.br/.

## Appendix Supplementary materials

Image, application 1
