# Supplementary material for: Risk of mortality for small newborns in Brazil, 2011-2018: A national birth cohort study of 17.6 million records from routine register-based linked data
Source: Lancet Reg Health Am. 2021 Aug 20;3:100045. doi: 10.1016/j.lana.2021.100045 (PMC8591743; doi:10.1016/j.lana.2021.100045)
Supplement: Supplementary file 1 [file mmc1.docx]

**Caption for supplementary material**

**Risk of mortality for small newborns in Brazil, 2011-2018: A national birth cohort study of 17.6 million records from routine register-based linked data**

Enny S. Paixao, Ph.D*^1,2^, Hannah Blencowe, MD, Ph.D^2^, Ila Rocha Falcao Ph.D^1,3^, Eric O Ohuma, Ph.D^2^, Aline dos Santos Rocha^1,3^, Flávia Jôse Oliveira Alves^1,4^, Maria da Conceição N. Costa, MD, Ph.D^1,4^, [Lorena Suárez-Idueta](https://www.scirp.org/journal/articles.aspx?searchcode=Lorena++Su%c3%a1rez-Idueta&searchfield=authors&page=1)^5^, Naiá Ortelan Ph.D^1^, Liam Smeeth, MD, Ph.D^2^, Laura C. Rodrigues MD, Ph.D^1,2^, Joy E Lawn, MB BS, Ph.D^2^, Marcia Furquim de Almeida, MD, Ph.D^6^, Maria Yury Ichihara, MD, PhD, Rita de Cássia Ribeiro Silva, Ph.D^1,3,^, Maria Gloria Teixeira MD, Ph.D^1,4^, Mauricio L. Barreto, MD, Ph.D^2,3^

**Table of contents**

**Table S1: Characteristics of 17,646,115 delivering live birth by phenotypes in Brazil from 2011-2018-------------------------2-3**

**Table S2: Characteristics of individuals excluded from the study in Brazil from 2011-2018 ---------------------4**

Table S3: Mortality risk by age group associated with the small vulnerable newborn phenotypes, excluding newborn with congenital abnormalities, Brazil 2011-2018________________________________________________5

**S4: Resumo---------------------------------------------------------------------------------------------6**

**Table S1: Characteristics of 17,646,115 delivering live birth by phenotypes in Brazil from 2011-2018.**

| **Characteristics** | **AGA+T+NBW** | | **SGA+T+NBW** | | **AGA+PT+NBW** | | **AGA+PT+LBW** | | **SGA+T+LBW** | | **SGA+PT+LBW** | | **AGA+T+LBW** | | **Total births** |
| --- | --- | --- | --- | --- | --- | --- | --- | --- | --- | --- | --- | --- | --- | --- | --- |
|  | **N** | **%** | **N** | **%** | **N** | **%** | **N** | **%** | **N** | **%** | **N** | **%** | **N** | **%** |  |
| **Maternal Age (Years)** |  |  |  |  |  |  |  |  |  |  |  |  |  |  |  |
| < 20 years | 2,592,357 | 79.3 | 206,746 | 6.3 | 136,486 | 4.2 | 156,088 | 4.8 | 129,702 | 4.0 | 28,720 | 0.9 | 17,829 | 0.5 | 3,267,928 |
| 20-35 | 10,085,172 | 82.9 | 539,369 | 4.4 | 440,546 | 3.6 | 511,492 | 4.2 | 400,601 | 3.3 | 117,949 | 1.0 | 63,717 | 0.5 | 12,158,846 |
| >35 | 1,767,728 | 79.7 | 79,917 | 3.6 | 101,567 | 4.6 | 132,786 | 6.0 | 86,402 | 3.9 | 35,532 | 1.6 | 15,241 | 0.7 | 2,219,173 |
| **Maternal ethnicity** |  |  |  |  |  |  |  |  |  |  |  |  |  |  |  |
| White | 5,251,965 | 82.6 | 230,596 | 3.6 | 257,119 | 4.0 | 315,737 | 5.0 | 196,221 | 3.1 | 69,086 | 1.1 | 38,508 | 0.6 | 6,359,232 |
| Black | 761,388 | 79.8 | 51,541 | 5.4 | 35,175 | 3.7 | 47,652 | 5.0 | 40,139 | 4.2 | 12,171 | 1.3 | 5,937 | 0.6 | 954,003 |
| Asian | 58,165 | 82.1 | 2,940 | 4.1 | 2,711 | 3.8 | 3,603 | 5.1 | 2,309 | 3.3 | 687 | 1.0 | 446 | 0.6 | 70,861 |
| Mixed race | 7,700,328 | 81.7 | 487,011 | 5.2 | 350,464 | 3.7 | 397,853 | 4.2 | 344,674 | 3.7 | 91,471 | 1.0 | 47,970 | 0.5 | 9,419,771 |
| Indigenous | 98,233 | 77.8 | 9,774 | 7.7 | 6,536 | 5.2 | 3,887 | 3.1 | 6,434 | 5.1 | 884 | 0.7 | 479 | 0.4 | 126,227 |
| **Marital status** |  |  |  |  |  |  |  |  |  |  |  |  |  |  |  |
| Single | 5,977,909 | 81.0 | 386,038 | 5.2 | 279,171 | 3.8 | 344,154 | 4.7 | 279,789 | 3.8 | 76,292 | 1.0 | 40,884 | 0.6 | 7,384,237 |
| Married/union | 8,132,186 | 82.6 | 421,654 | 4.3 | 382,603 | 3.9 | 437,160 | 4.4 | 322,404 | 3.3 | 101,298 | 1.0 | 53,634 | 0.5 | 9,850,939 |
| Widow | 24,936 | 80.6 | 1,427 | 4.6 | 1,286 | 4.2 | 1,510 | 4.9 | 1,193 | 3.9 | 365 | 1.2 | 202 | 0.7 | 30,919 |
| Divorced | 156,961 | 81.6 | 6,819 | 3.5 | 8,349 | 4.3 | 10,290 | 5.3 | 6,351 | 3.3 | 2,462 | 1.3 | 1,226 | 0.6 | 192,458 |
| **Maternal education** |  |  |  |  |  |  |  |  |  |  |  |  |  |  |  |
| None | 72,304 | 74.6 | 8,096 | 8.4 | 4,268 | 4.4 | 4,159 | 4.3 | 6,448 | 6.7 | 1,183 | 1.2 | 469 | 0.5 | 96,927 |
| 1 - 3 years | 403,719 | 78.4 | 33,692 | 6.5 | 21,526 | 4.2 | 21,992 | 4.3 | 25,665 | 5.0 | 5,808 | 1.1 | 2,589 | 0.5 | 514,991 |
| 4 - 7 years | 2,646,788 | 79.9 | 198,230 | 6.0 | 132,183 | 4.0 | 147,302 | 4.4 | 139,018 | 4.2 | 33,333 | 1.0 | 17,833 | 0.5 | 3,314,687 |
| 8-12 years | 8,436,419 | 82.3 | 480,962 | 4.7 | 378,559 | 3.7 | 450,330 | 4.4 | 347,136 | 3.4 | 101,478 | 1.0 | 54,376 | 0.5 | 10,249,260 |
| ≥ 12 years | 2,683,797 | 83.3 | 90,977 | 2.8 | 132,585 | 4.1 | 167,177 | 5.2 | 88,675 | 2.8 | 38,013 | 1.2 | 20,341 | 0.6 | 3,221,565 |
| **Multiplus fetuses** |  |  |  |  |  |  |  |  |  |  |  |  |  |  |  |
| Yes | 108,573 | 25.8 | 13,139 | 3.1 | 43,444 | 10.3 | 152,019 | 36.1 | 56,225 | 13.3 | 35,397 | 8.4 | 12,658 | 3.0 | 421,455 |
| None | 14,316,826 | 83.2 | 811,646 | 4.7 | 634,197 | 3.7 | 647,396 | 3.8 | 559,626 | 3.3 | 146,574 | 0.9 | 84,029 | 0.5 | 17,200,294 |
| **Sex of newborn** |  |  |  |  |  |  |  |  |  |  |  |  |  |  |  |
| Male | 7,378,763 | 81.7 | 459,820 | 5.1 | 394,752 | 4.4 | 406,174 | 4.5 | 261,862 | 2.9 | 89,047 | 1.0 | 35,894 | 0.4 | 9,026,312 |
| Female | 7,066,610 | 82.0 | 366,233 | 4.2 | 283,860 | 3.3 | 394,194 | 4.6 | 354,858 | 4.1 | 93,155 | 1.1 | 60,893 | 0.7 | 8,619,803 |
| **Congenital abnormalities** |  |  |  |  |  |  |  |  |  |  |  |  |  |  |  |
| Yes | 91,520 | 62.0 | 7,789 | 5.3 | 7,695 | 5.2 | 17,215 | 11.7 | 13,853 | 9.4 | 7,958 | 5.4 | 1,487 | 1.0 | 147,517 |
| None | 14,022,232 | 82.0 | 797,316 | 4.7 | 655,054 | 3.8 | 764,837 | 4.5 | 587,489 | 3.4 | 169,772 | 1.0 | 93,254 | 0.5 | 17,089,954 |
| **Mode of delivery** |  |  |  |  |  |  |  |  |  |  |  |  |  |  |  |
| Caesarean section | 7,947,149 | 82.0 | 373,835 | 3.9 | 368,672 | 3.8 | 479,092 | 4.9 | 334,410 | 3.5 | 132,313 | 1.4 | 56,744 | 0.6 | 9,692,215 |
| Vaginal Delivery | 6,482,878 | 81.7 | 451,167 | 5.7 | 309,208 | 3.9 | 320,572 | 4.0 | 281,575 | 3.5 | 49,710 | 0.6 | 39,954 | 0.5 | 7,935,064 |

**Table S2: Characteristics of individuals excluded from the study in Brazil from 2011-2018.**

| **Characteristics** | **Study Sample** | | **Excluded due to missing information on gestational age at birth and birth weight or newborn sex** | | **Excluded due to implausible values for birth weight and gestational age** | | **Excluded due <24 and more than 42 Weeks** | |
| --- | --- | --- | --- | --- | --- | --- | --- | --- |
|  | **N** | **(%)** | **N** | **(%)** | **N** | **(%)** | **N** | **(%)** |
| **Age of the mother (Years)** |  |  |  |  |  |  |  |  |
| < 20 years | 3,267,928 | 18.52 | 396,824 | 19.41 | 11,843 | 23.46 | 59,465 | 23.9 |
| 20-35 | 12,158,846 | 68.9 | 1,417,773 | 69.35 | 34,284 | 67.91 | 168,188 | 67.59 |
| >35 | 2,219,173 | 12.58 | 229,813 | 11.24 | 4,360 | 8.63 | 21,175 | 8.51 |
| Missing | 168 |  | 6 |  | 1 |  | 4 |  |
| **Maternal ethnicity** |  |  |  |  |  |  |  |  |
| White | 6,359,232 | 37.56 | 112,168 | 19.03 | 10,833 | 22.79 | 56,830 | 23.92 |
| Black | 954,003 | 5.63 | 30,430 | 5.16 | 2,903 | 6.11 | 14,342 | 6.04 |
| Asian | 70,861 | 0.42 | 1,684 | 0.29 | 210 | 0.44 | 756 | 0.32 |
| Mixed race | 9,419,771 | 55.64 | 430,366 | 73 | 32,990 | 69.42 | 162,777 | 68.5 |
| Indigenous | 126,227 | 0.75 | 14,861 | 2.52 | 589 | 1.24 | 2,914 | 1.22 |
| Missing | 716,021 |  | 1,454,907 |  | 2,963 |  | 11,213 |  |
| **Marital status** |  |  |  |  |  |  |  |  |
| Single | 7,384,237 | 42.3 | 1,109,911 | 55.54 | 21,668 | 43.74 | 108,897 | 44.51 |
| Married/union | 9,850,939 | 56.42 | 861,169 | 43.09 | 27,389 | 55.29 | 133,543 | 54.58 |
| Widow | 30,919 | 0.18 | 4,423 | 0.22 | 94 | 0.19 | 471 | 0.19 |
| Divorced | 192,458 | 1.1 | 22,864 | 1.15 | 384 | 0.78 | 1,773 | 0.72 |
| Missing | 187,562 |  | 46,049 |  | 953 |  | 4,148 |  |
| **Maternal education** |  |  |  |  |  |  |  |  |
| None | 96,927 | 0.56 | 27,187 | 1.39 | 639 | 1.3 | 2,869 | 1.18 |
| 1 - 3 years | 514,991 | 2.96 | 110,483 | 5.64 | 3,035 | 6.18 | 13,893 | 5.71 |
| 4 - 7 years | 3,314,687 | 19.05 | 492,852 | 25.16 | 14,208 | 28.94 | 69,732 | 28.67 |
| 8-12 years | 10,249,260 | 58.91 | 990,350 | 50.57 | 26,928 | 54.85 | 137,116 | 56.38 |
| ≥ 12 years | 3,221,565 | 18.52 | 337,673 | 17.24 | 4,285 | 8.73 | 19,588 | 8.06 |
| Missing | 248,685 |  | 85,871 |  | 1,393 |  | 5,634 |  |
| **Multiples fetus** |  |  |  |  |  |  |  |  |
| Yes | 421,455 | 2.39 | 37,873 | 1.86 | 1,200 | 2.38 | 5,152 | 2.08 |
| None | 17,200,294 | 97.61 | 1,994,366 | 98.14 | 49,129 | 97.62 | 242,983 | 97.92 |
| Missing | 24,366 |  | 12,177 |  | 159 |  | 697 |  |
| **Sex of newborn** |  |  |  |  |  |  |  |  |
| Male | 9,026,312 | 51.15 | 1,045,168 | 51.23 | 25,096 | 49.8 | 125,439 | 50.41 |
| Female | 8,619,803 | 48.85 | 995,129 | 48.77 | 25,294 | 50.2 | 123,393 | 49.59 |
| Missing | 0 |  | 4,119 |  | 98 |  | 0 |  |
| **Congenital abnormalities** |  |  |  |  |  |  |  |  |
| Yes | 147,517 | 0.86 | 17,141 | 0.88 | 428 | 0.88 | 1,841 | 0.77 |
| None | 17,089,954 | 99.14 | 1,923,876 | 99.12 | 48,089 | 99.12 | 238,135 | 99.23 |
| Missing | 408,644 |  | 103,399 |  | 1,971 |  | 8,856 |  |
| **Mode of delivery** |  |  |  |  |  |  |  |  |
| Caesarean section | 9,692,215 | 54.98 | 1,091,964 | 53.71 | 22,813 | 45.31 | 108,702 | 43.77 |
| Vaginal Delivery | 7,935,064 | 45.02 | 941,247 | 46.29 | 27,533 | 54.69 | 139,618 | 56.23 |
| Missing | 18,836 |  | 11,205 |  | 142 |  | 512 |  |

|  |  | **Neonatal mortality** | | |  | **Infant mortality** | | |  | **Under five mortality** | | |
| --- | --- | --- | --- | --- | --- | --- | --- | --- | --- | --- | --- | --- |
|  |  |  | HR (95%CI) | |  |  | HR (95%CI) | |  |  | HR (95%CI) | |
|  | Number of deaths | Deaths/1000 PY | Unadjusted | Adjusted | Number of deaths | Deaths/1000 PY | Unadjusted | Adjusted | Number of deaths | Deaths/1000 PY | Unadjusted | Adjusted |
| **Term+AGA+NBW** | 21824 | 20.7  (20.4-21.0) | Ref | Ref | 44401 | 3.3  (3.3-3.3) | Ref | Ref | 57246 | 1.2  (1.2-1.2) | Ref | Ref |
| **Term+SGA+NBW** | 2976 | 49.6  (47.8-51.4) | 2.3  (2.3-2.5) | 2.2  (2.1-2.3) | 5534 | 7.3  (7.1-7.5) | 2.2  (2.1-2.3) | 2.0  (1.9-2.1) | 6778 | 2.5  (2.4-2.5) | 2.1  (2.0-2.1) | 1.9  (1.8-1.9) |
| **Preterm+AGA+NBW** | 3092 | 62.8  (60.7-65.1) | 3.0  (2.9-3.1) | 3.0  (2.9-3.1) | 5125 | 8.2  (8.0-8.4) | 2.5  (2.4-2.6) | 2.5  (2.4-2.5) | 5947 | 2.6  (2.6-2.7) | 2.2  (2.2-2.3) | 2.2  (2.1-2.2) |
| **Term+AGA+LBW** | 359 | 51.3  (46.3-56.9) | 2.5  (2.2-2.7) | 2.7  (2.4-3.0) | 717 | 8.1  (7.6-8.7) | 2.5  (2.3-2.6) | 2.7  (2.4-2.7) | 864 | 2.8  (2.6-2.9) | 2.3  (2.1-2.4) | 2.4  (2.3-2.6) |
| **Term+SGA+LBW** | 6679 | 151.9  (148.3-155.6) | 7.3  (7.1-7.5) | 7.2  (7.0-7.4) | 10876 | 19.6  (19.2-20.0) | 5.9  (5.8-6.1) | 5.7  (5.6-5.9) | 12132 | 6.1  (6.0-6.2) | 5.1  (5.0-5.2) | 4.9  (4.8-5.0) |
| **Preterm+AGA+LBW** | 47102 | 859.8  (852.1-867.6) | 40.7  (40.0-41.3) | 43.8  (43.1-44.5) | 58735 | 86.7  (86.0-87.4) | 25.5  (25.2-25.8) | 27.2  (26.9-27.6) | 60409 | 25.4  (25.2-25.7) | 20.3  (20.0-20.5) | 21.6  (21.4-21.9) |
| **Preterm+SGA+LBW** | 18446 | 1572.8  (1550.3-1595.7) | 72.9  (71.5-74.4) | 81.8  (80.2-83.6) | 22737 | 159.9  (157.8-162.0) | 45.8  (45.0-46.5) | 50.5  (49.6-51.3) | 23271 | 46.5  (45.9-47.1) | 36.3  (35.7-36.8) | 39.9  (39.2-40.5) |
|  |  |  |  |  |  |  |  |  |  |  |  |  |

**Table S3: Mortality risk by age group associated with the small vulnerable newborn phenotypes, excluding newborn with congenital abnormalities, Brazil 2011-2018**

**S4: Resumo**

**Introdução:** Nascimento prematuro (<37 semanas), baixo peso ao nascer (BPN, <2500g) e pequeno para a idade gestacional (PIG, <percentil 10 do peso ao nascer para a idade gestacional e sexo) são marcadores de vulnerabilidade do recém-nascido com alto risco de mortalidade. Neste estudo a prevalência de fenótipos combinando esses três marcadores foi estimada e o risco de mortalidade associado a eles quantificado.

**Métodos:** Foi realizado um estudo de coorte de base populacional,nutilizando dados vinculados de registros de rotina sobre todos os nascimentos e óbitos no Brasil de 1º de janeiro de 2011 a 31 de dezembro de 2018. Foi estimada a prevalência de prematuros, BPN e PIG individualmente e para fenótipos combinando estas características. A mortalidade neonatal precoce, neonatal tardio, neonatal, pós-neonatal, infantil, 1-4 anos e menores de cinco anos foi quantificada usando taxas de mortalidade e as razões de risco(HRs) com intervalo de confiança xom 95% (IC 95%) foram estimadas através de modelos de riscos proporcionais de Cox.

**Resultados:** Foram incluídos 17.646.115 de nascidos vivos. As prevalências de prematuridade, BPN e PIG foram de 9,4%, 9,6% e 9,2%, respectivamente. O risco de mortalidade neonatal foi 16 vezes (HR = 15,9; IC 95%: 15,7–16,1) maior para prematuros em comparação com termos, 3 vezes maior (HR = 3,4; (IC 95%: 3,3–3,4) para PIG em comparação com o adequado para a idade gestacional (AIG) e 25 vezes maior para o BPN (HR = 25,8; (IC 95%: 25,5-26,1) em comparação com o peso normal ao nascer. 18% de todos os nascidos vivos foram incluídos em um dos fenótipos estudados. Desses 8,2% eram PIG-termo (4,7% peso normal, 3,5% BPN), 0,6% AIG-termo-BPN, 8,3% AGA-pretermo (3,8% peso normal, 4,5% BPN) e 1,0% prematuro- PIG-BPN. Em comparação aos termo-AGA-peso normal, o maior risco de mortalidade foi para os fenótipos de BPN-prematuro: AGA-BPN-pretermo (HR = 36,2 (IC 95% 35,6-36,8) e SGA-BPN-pretermo (HR = 62,0 (IC 95% 60,8-63,2). O risco aumentado de mortalidade associado a fenótipos de recém-nascidos vulneráveis foi maior no primeiro mês de vida, com atenuado, mas maior risco continuado no pós-neonatal e de 1 a 4 anos.

**Interpretação:** Nossos achados apoiam a importância de fenótipos mais detalhados para identificar os de maior risco. Assim, contribuem para intensificar políticas de atenção e cuidado no nível individual, avançando na pesquisa, especialmente para prevenção, e acelerando o progresso em direção a metas globais, como os Objetivos de Desenvolvimento Sustentável.

**Financiamento**: Wellcome Trust
